# Impact on Human Health of *Salmonella* spp. and Their Lipopolysaccharides: Possible Therapeutic Role and Asymptomatic Presence Consequences

**DOI:** 10.3390/ijms252211868

**Published:** 2024-11-05

**Authors:** Mateusz Mikołajczyk, Dagmara Złotkowska, Anita Mikołajczyk

**Affiliations:** 1 Division of Medicine and Dentistry, Medical University of Warsaw, 02-091 Warsaw, Poland; matmateusza@gmail.com; 2 Department of Food Immunology and Microbiology, Polish Academy of Sciences, 10-748 Olsztyn, Poland; d.zlotkowska@pan.olsztyn.pl; 3 Department of Psychology and Sociology of Health and Public Health, Collegium Medicum, University of Warmia and Mazury in Olsztyn, 10-719 Olsztyn, Poland

**Keywords:** *Salmonella*, LPS, asymptomatic carrier state, oncogenic and oncolytic *Salmonella*, asymptomatic LPS, cancer, serotype diverse activity, biosafety, unknown long-term consequences

## Abstract

Epidemiologically, one of the most important concerns associated with introducing *Salmonella* spp. into the environment and food chain is the presence of asymptomatic carriers. The oncogenic and oncolytic activity of *Salmonella* and their lipopolysaccharides (LPSs) is important and research on this topic is needed. Even a single asymptomatic dose of the *S.* Enteritidis LPS (a dose that has not caused any symptoms of illness) in in vivo studies induces the dysregulation of selected cells and bioactive substances of the nervous, immune, and endocrine systems. LPSs from different species, and even LPSs derived from different serotypes of one species, can define different biological activities. The activity of low doses of LPSs derived from three different *Salmonella* serotypes (*S*. Enteritidis, *S.* Typhimurium, and *S.* Minnesota) affects the neurochemistry of neurons differently in in vitro studies. Studies on lipopolysaccharides from different *Salmonella* serotypes do not consider the diversity of their activity. The presence of an LPS from *S*. Enteritidis in the body, even in amounts that do not induce any symptoms of illness, may lead to unknown long-term consequences associated with its action on the cells and biologically active substances of the human body. These conclusions should be important for both research strategies and the pharmaceutical industry &.

## 1. Introduction

The ability of pathogens to infect higher taxa organisms is still a significant problem, and the existence of asymptomatic carriers poses a major epidemiological threat. Non-typhoidal and typhoidal *Salmonella* are common infectious enteric pathogens with the ability to persist in the host without causing any clinical symptoms of infection. *Salmonella* represents one of the most common causes of food-borne toxicoinfections of bacterial origin. Despite efforts to address it, salmonellosis, which is an infectious disease primarily transmitted through food of animal origin, continues to pose a significant public health concern. Epidemiologically, one of the most important concerns associated with the introduction of pathogenic bacteria such as *Salmonella* spp. into the food chain is the existence of asymptomatic carriers [1]. It is crucial to understand the biology of *Salmonella* and the response of both asymptomatic and symptomatic hosts of *Salmonella* spp. Thus, any emerging research into the pathogens responsible for inducing an asymptomatic carrier state is of great value.

Unfortunately, the role of microorganisms capable of causing latent infections and the mechanism underlying the process of the *Salmonella* asymptomatic carrier state, as well as the influence of their endotoxins (released after the death of a bacterial cell) on the body, have not been fully explained [2]. There is limited knowledge regarding *Salmonella’s* ability to survive for extended periods inside the body while evading detection by the immune system. The specific virulence factors required for persistent *Salmonella* infection remain unclear. Much effort has been concentrated on the *Salmonella* pathogenicity islands 1 and 2 (SPI1 and SPI2, respectively), but the extent of their contribution to persistent infection is still uncertain. A recent study [3] determined mutations in *barA* and *sirA* genes, which control the expression of virulence factors (including those located on *Salmonella* SPI-1) during persistent infections in humans. Perhaps these genetic changes can play an important role in host–pathogen interactions establishing persistent, long-term infections with non-typhoidal *Salmonella*.

Transcriptional and proteomic changes can also play a role in the persistence of *Salmonella*. However, the specific factors that trigger these changes have not been fully determined [4]. The reasons why the immune system fails to recognize *Salmonella* and the methods by which *Salmonella* manages to evade the body’s immune response are still unknown. *S.* Typhimurium remains dormant inside enterocytes within a unique vesicular compartment, which is distinct from the conventional modified phagosome known as the *Salmonella*-containing vacuole. These dormant epithelial *S.* Typhimurium with restricted access to the extracellular nutrients are viable and persist within host cells for a prolonged period (at least seven days under test conditions). The dormant state mechanism in enterocytes is distinct from that in fibroblasts and macrophages. Dormancy and delayed expression of SPI-2 virulence factors allow *S*. Typhimurium to evade cellular immunity during the early invasion. SPI-2 reactivated from dormancy helps *S.* Typhimurium survive in the gut lumen [5].

The fact that some pathogens can remain dormant for a certain time may also be related to the phenomenon of immune tolerance and/or the issue of the level of microorganisms and their toxins in the body. *Salmonella* colonization may be transient or carried out for a prolonged period. Long-term colonization by *Salmonella* in its host is referred to as “persistence”. In this state, the pathogen adopts a low metabolic state, which may also be due to the selective pressure of the host’s immune resistance. A persistent state and close association between *Salmonella* and autoimmune diseases, gallbladder carcinoma, and colorectal cancer highlight the need to invest efforts in preventing, diagnosing, and treating *Salmonella*-related diseases [6,7]. As the number of *Salmonella* infections and multidrug resistance increase, there is a real need for a vaccine strategy [8]. One of *Salmonella’s* most significant disease-causing factors is the endotoxin lipopolysaccharide (LPS). Endotoxins induce a range of biological activities at the systemic level that are capable of causing both positive and negative pathological changes in many tissues.

The article aims to highlight the diverse biological activity of LPSs, which are dependent on the serotypes of *Salmonella.* It also aims to draw attention to the unknown long-term consequences of asymptomatic LPS use in the context of oncogenic and oncolytic *Salmonella* activity. The literature review for this article included an extensive search across various scientific databases. After a thorough examination of full texts for congruity, methodological relevance, and credibility, the appropriate data were selected. The analysis provided a deeper understanding of the review topic, which led to the formulation of conclusions.

## 2. Inflammatory Activity of *Salmonella* spp. and Their LPSs

It is known that *Salmonella* spp. and their LPSs, as well as metabolites of LPSs (such as 3-hydroxy fatty acids), can persist in the human body for many years [9,10]. Niehaus and Lange [11] and Niehaus [9] reported a case of a laboratory worker who experienced accidental exposure to *S.* Minnesota, which initially caused inflammation and, in consequence, probably the symptoms of polyneuropathy, encephalopathy, and parkinsonism. After 14 years following the incident, despite various therapies implemented, he still tested positive for the presence of the *S.* Minnesota-derived endotoxin. The circulating LPS is cleared from the circulation very fast. Still, the remaining ~20% of the LPS can be bound to immune cells (for example, monocytes, tissue macrophages, neutrophils, and platelets) and can be involved in signaling pathways [12].

It is widely known that a high dose of LPS exhibits proinflammatory activity and plays a role in sepsis [13]. Mohammadi et al. [14] observed that LPSs are strongly implicated in the pathogenesis of critical illness neuropathy. LPS may be potentially life-threatening, although it may also exert a beneficial effect through the stimulation of the immune system. The activity of LPS, which has been most thoroughly examined, consists primarily of the stimulation of the host cells to release a variety of inflammatory mediators. The interaction of LPS with the receptors found on the surface of monocytes, lymphocytes, and vascular endothelial cells induces the release of proinflammatory cytokines, such as tumor necrosis factor α (TNF-α) and interleukin 6 (IL-6), as well as acute phase proteins such as haptoglobin (Hp). The immediate effect of LPS activity is its ability to induce septic shock, both in people and animals, often leading to death.

A high dose of LPS, usually administered directly into the substantia nigra, has long been used in experimental animal models to mimic the symptoms of Parkinson’s disease (PD) in humans [15,16,17,18,19]. These models utilize the LPS’s ability to activate microglia cells, leading to the release of inflammatory mediators. Inflammatory processes and the TLR4 signaling pathway are involved in neuroinflammation and neurodegeneration in PD. However, a direct causal link between TLR4 and PD pathology requires further study in animals [20]. Moreover, intestinal barrier dysfunction can be connected with PD, especially since LPS can alter gut permeability [21,22]. Recently, there has been a growing focus on studying the effects of both pathogenic and non-pathogenic bacteria, as well as the gut barrier on the etiology and pathogenesis or clinical course of mental disorders like depression and neurodegenerative diseases such as Alzheimer’s disease (AD) and PD. Numerous studies [23,24,25] have demonstrated that LPS proinflammatory activity also plays an important role in AD, amyotrophic lateral sclerosis, and in mental disorders with cognitive function impairment. Regrettably, the source of LPS, despite its significance, was occasionally omitted by some authors. In only one of the mentioned studies did the authors report the sources of *Salmonella* spp. (the species along with the serotype). The LPS from *S.* Minnesota injected into the striatum of rats caused the progressive degeneration of the dopamine nigrostriatal system, accompanied by motor impairments [23]. It is crucial for publications to consistently report the source of the lipopolysaccharide used in the research, including the specific serotype. Unfortunately, some articles, including articles with samples from humans, lack key information about a particular type of LPS [26]. LPS may play a significant role in some neurodegenerative, oncological, and metabolic disorders, not only in rodents but also in people [27,28,29,30,31,32,33]. The mechanism through which LPS may be associated with the abovementioned processes is not understood. LPS is probably associated with the neuropathology of AD in people owing to the fact that it might be capable of crossing the blood–brain barrier [34]. However, the manner in which LPS administered peripherally affects the central nervous system is still unclear [35,36,37,38].

For chronic inflammation-mediated diseases, the role of infectious agents is being increasingly emphasized. It is crucial to identify the factors strongly associated with these diseases, as they can significantly contribute to reducing the associated morbidity. The virulence factors of *Salmonella*, inflammation pathways, and individual susceptibility contribute to the pathogenesis of reactive arthritis (ReA) and inflammatory bowel disease (IBD). Microbial infection, such as *Salmonella* infection leading to the impaired immune system, ultimately results in intestinal or extraintestinal autoimmune diseases such as IBD and ReA, respectively [6,7]. *Salmonella* infection, like genetic and environmental factors, contributes to alterations and the deregulation of immune responses.

## 3. Oncogenic Activity of *Salmonella* spp. and Their LPSs

**Figure 1:**
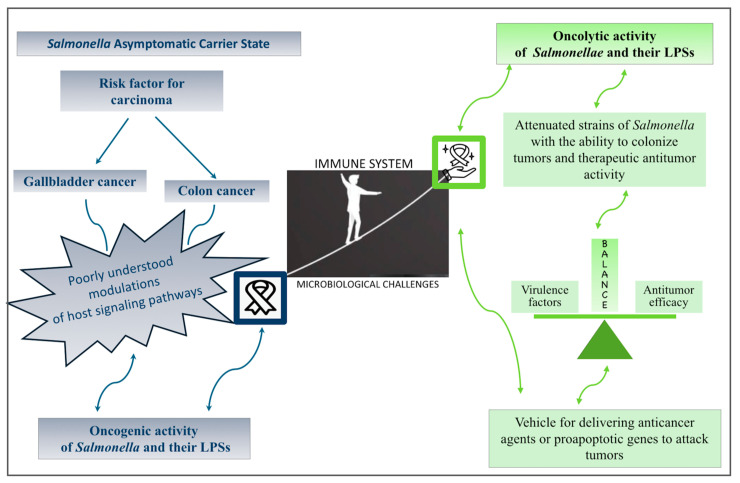
The role of *Salmonella* in cancer treatment and in the development and progression of cancer. The *Salmonella* carrier state represents a risk factor for gallbladder cancer and colon cancer. *Salmonella* strains have been engineered to be an effective cancer therapy tool.

*Salmonella* infection, encompassing diverse serotypes (for example, *S.* Typhi, *S.* Typhimurium, and *S.* Enteritidis), is also linked to colon and gallbladder cancer development [6,39,40]. The carrier state for both non-typhoidal and typhoidal *Salmonella* represents a risk factor for gallbladder cancer (Figure 1). This is confirmed by a study in which the *Salmonella* ribosomal genes, genes involved in metabolism, and those related to the toxin–antitoxin system (*23S rRNA*, *t0254*, *t2612*, *t4108 rrlA*, *rrlB*, *rrlC*, *rrlD*, *rrlE*, *rrlG*, *rrsH*, *tuf*, *dkgB*, *pduC*, *rpoC*, and *yjgF)* were identified after the whole-exome examination of the primary gallbladder tumor samples. It is interesting that *S.* Typhi, *S.* Paratyphi, *S.* Typhimurium, and *S.* Choleraesuis were found in the tumors of gallbladder tissues as well as adjacent normal tissues [41]. Other studies [42,43,44] have expanded the association of *Salmonella* with gallbladder cancer and colorectal carcinoma, although further study is required to establish the causality of infection in relation to these diseases in a more comprehensive manner [6].

*Salmonella* bacteria can cause changes in organisms, promoting carcinogenesis by stimulating a host response. *Salmonella* can promote malignant transformation in murine gallbladder organoids and fibroblasts by the activation of the AKT and MAP kinase pathways [45]. *Salmonella* modulation of host signaling pathways like the AKT-MAP kinase pathway can play a role both in gallbladder carcinoma and colon cancer [7,46]. *Salmonella* can exploit effector proteins to inhibit (e.g., IpaJ, SptP, AvrA, and SpvC) or stimulate (e.g., SopE and SteC) MAPK cascades [47]. *Salmonella* uses various mechanisms to survive in host cells and promote colon cancer. *Salmonella* secretes a range of effector proteins, such as AvrA, into the host cells via the SPI-1 type III secretion system (T3SS) [48]. AvrA is a key effector in colon cancer development. AvrA from *S.* Enteritidis inhibited autophagy to promote bacterial survival in the host [49].

These mechanisms of cancer progression can also be linked to bacterial biofilms. The LPS of *S.* Typhi facilitates the formation of biofilms. A conductive environment for bacterial adhesion in the gallbladder supports the growth of *Salmonella* on gallstone surfaces. *S.* Typhi invades the mucosal surface of the gallbladder and modulates the expression of proteins crucial for biofilm formation, releasing carcinogenic agents like bacterial glucuronidase, nitroso compounds, and toxin complexes to promote DNA damage and causing genomic instability that may result in gallbladder cancer [50]. Understanding all of these processes, including the mechanisms by which biofilms impact oncogenesis, may optimize treatment strategies. Moreover, the association between the microbiota, the host, and pathogenic bacteria, e.g., *Salmonella*, is important. *Salmonella* can modify the genomic, taxonomic, and functional traits of the gastrointestinal microbiota. Further research is needed to better understand the interaction between the gut microbiota and various *Salmonella* serotypes and their persistent state [51].

Aspects that the immune system primarily controls to combat microbiological challenges need a better understanding. Similar but different immunological mechanisms can also remove damaged and aberrant cells, including cancer cells, to induce long-term cures. *Salmonella* may contribute to the development and progression of cancer, affect the complications associated with the neoplastic process, and promote anticancer drug therapy [52].

## 4. Oncolytic Activity of *Salmonella* spp. and Their LPSs

*Salmonella*, as an intracellular pathogen, exhibits the intrinsic therapeutic efficacy and specificity of tumor colonization. A recent study demonstrated that therapeutic efficacy against murine cells of colon cancer does not require bacterial viability and can be induced by LPS from *S.* Typhi [53]. LPS exhibits antitumor activity, but its effective doses for damaging cancer cells are poorly tolerated by organisms. Various strategies have been developed to improve LPS tolerance. A recent preclinical study [54] supports the safety of the intravenous administration of chemically detoxified monophosphorylated LPS. This LPS formulated in liposomes has antitumor activity in mice models.

*Salmonella* bacteria have demonstrated potential for use in cancer therapy [55,56,57]. *Salmonella’s* engineered or attenuated strains have been designed to target various solid cancers, making them ideal vectors for delivering and expressing immunostimulatory agents [56,58]. Attenuated *S.* Typhimurium is able to naturally accumulate and replicate in a wide variety of solid tumors [55]. The construction of highly attenuated strains of *Salmonella* with the ability to colonize tumors and therapeutic activity is challenging. The attenuated VNP20009 strain of *S.* Typhimurium has been extensively researched for its ability to target solid tumors. This strain can be safely administered to patients due to the deletion of virulence genes required for lipid A and adenine synthesis. The strain was evaluated in a phase 1 clinical trial for the treatment of nonresponsive metastatic melanoma [59]. However, *S.* Typhimurium VNP20009 was unable to effectively colonize tumors, possibly due to over-attenuation, leading to a lack of significant antitumor effects [60]. Some studies suggest that the antitumor effect of *Salmonella* is related to its virulence factors [56,61,62]. It seems that finding the best equilibrium between the antitumor efficacy and toxicity of *Salmonella* (the balance of therapeutic benefits and over-attenuation) can help to develop anticancer therapy (Figure 1).

Cytolysin A (ClyA) is a bacterial toxin native to *S.* Typhimurium. Although it was previously considered to be poorly immunogenic, a recent report [63] showed that ClyA could enhance LPS-induced IL-1β secretion in human macrophages through TLR4 and NLRP3 signaling. This finding suggests that ClyA could potentially be utilized in the treatment of colon tumor cells. This toxin, due to its pore-forming mechanism, can permeate the neutrophilic barrier, destroy cancer stromal cells and cancer cells in mouse models of human pancreatic cancer, and cause the infiltration of immune cells (neutrophils and macrophages) into tumors. The attenuated *S.* Typhimurium engineered to express ClyA colonized the tumor and exhibited oncolytic activity [64]. The attenuated *S.* Typhimurium engineered to express ClyA, when compared to VNP20009, also demonstrated the ability to induce higher levels of immune cell infiltration and release elevated levels of TNF-α, IL-1β, and other antitumor inflammatory factors in the colorectal cancer model. Additionally, this mutant strain exhibits high safety profiles in vivo, providing better conditions for combinational therapies [65,66].

The anticancer effectiveness of *Salmonella* in clinical trials can be attributed to the development of *S.* Typhimurium mutants with a high specificity for targeting tumors, the ability to penetrate deep tissues, low systemic toxicity, a balanced combination of virulence factors that stimulate the immune system, and significant attenuation linked with dose-dependent adverse effects [66]. Recent studies have shown that clinical trials involving attenuated *Salmonella* have demonstrated improved therapeutic effects when used in combination with other antitumor therapies. These treatments include the delivery of siRNA by attenuated *Salmonella* to cancerous tissue. This combined approach can work together to inhibit the expression of VEGF and PD-L1, and also contribute to an increase in T-cell infiltration in hepatocellular carcinoma tumors [67,68]. Moreover, the attenuated *Salmonella* carrying siRNA-PD-L1 could effectively enhance the antitumor effect of radiotherapy on hepatocellular carcinoma-bearing mice [69]. Cancer patients are unable to eliminate cancer cells through their immune system because the cancer cells can create various ways to evade the immune system, such as activating immune checkpoint molecules. Immune checkpoint inhibitors (ICIs) are utilized in the treatment of certain human cancers, but resistance to the PD-1/PD-L1 blockade hinders the effective use of ICIs and necessitates further research [70].

As described above, attenuated *Salmonella* can act as a vehicle for delivering anticancer agents or proapoptotic genes to attack tumors. Attenuated *Salmonella* strains can stimulate and enhance the host immune system to fight cancer. Moreover, *Salmonella* colonizing tumors can achieve oncolytic activity through a variety of pathways, including the induction of tumor cell death, inhibition of tumor angiogenesis, inhibition of tumor metastasis, or reduction in tumor drug resistance. A more in-depth exploration of the anticancer capabilities of oncolytic bacteria such as *Salmonella* is required.

## 5. Safety and Unknown Long-Term Consequences of Asymptomatic LPSs

*Salmonella* strains, for example, *S.* Typhimurium strains, have been engineered to enhance their safety for use in cancer therapy. It is crucial to find a balance between reducing the virulence of the bacteria and maintaining their therapeutic effectiveness. The complete elimination of bacterial virulence is not recommended as it could impact therapeutic benefits. When genetically modifying *Salmonellae*, it is important to consider both the health benefits and risks, including the elimination of bacterial resistance to antibiotics. Moreover, although no deaths have occurred in studies on volunteers, information about both the short-term and long-term side effects of *Salmonella* and their LPS constituents is necessary.

The peripheral administration of LPS is a widely used experimental model for inducing inflammation and sickness symptoms in both animals and humans. LPS administration in animals provides a suitable model for studying bacterial infections in humans [71]. It is important to note that rodents are less sensitive to LPS and require doses 10^6^ times greater (1–25 mg/kg) than those used in humans (2–8 ng/kg) to induce the release of proinflammatory cytokines. In behavior studies, the doses of LPS are lower (for example, in humans it is 0.4 ng/mL and in rodents it is 0.1 mg/kg body weight). LPS is typically administered intravenously (i.v.) in humans, but, when injected intraperitoneally (i.p.) in rodents, it results in about ten times lower circulating LPS concentration compared to i.v. administration. LPS doses administered in animals may vary depending on the different animal strains, animal age, and genetic predisposition [71,72].

**Figure 2:**
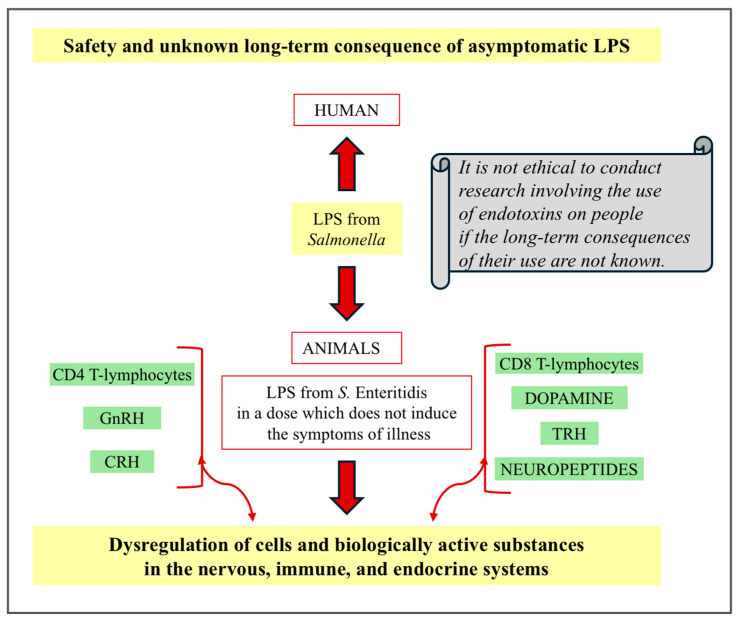
Dysregulation of cells and biologically active substances by asymptomatic LPS from *S.* Enteritidis in the animal model—unknown long-term consequences of LPSs used for humans (CRH-corticoliberin, corticotropin-releasing hormone; TRH-thyroliberin, thyrotropin-releasing hormone; GnRH-gonadoliberin, gonadotropin-releasing hormone).

Lipopolysaccharides are crucial for studying therapeutic development and understanding the immune system. The most commonly used LPSs in studies, both in humans and animals, come from *E. coli*. There are only a few studies available that have used the parenteral administration of unmodified *Salmonella*-derived LPS in in vivo animal studies [73,74,75,76,77,78,79,80,81,82,83]. LPSs from *Salmonella* Abortus equi were used in phase I/II clinical human trials many years ago. Intravenous administration doses (0.15 to 0.5 ng/kg in phase I and 0.4 ng/kg in phase II) of LPSs from *Salmonella* Abortus equi do not achieve antitumor activity both in colorectal and non-small-cell lung cancer [73,80]. LPSs derived from various bacteria have been administered many times to people in various experiments [71,72,84]. The studies focused primarily on the assessment of blood parameters and did not involve tissues due to their limited availability for biopsy or other tests. Therefore, the significance of some aspects of unknown consequences induced by LPS requires animal models. Sometimes, the domestic pig model is used to understand the processes occurring in the human body because pigs are phylogenetically closer to people than rodents. It is known that the systemic administration of LPS at low doses induces the production of proinflammatory cytokines that activate the immune system, resulting in disease symptoms. In an experimental model using domestic pigs, it was observed that the administration of LPS from *S.* Enteritidis at an asymptomatic dose (5 μg/kg b.w.) may have an effect on the nervous system and the immune system even seven days from administration of LPS [77]. LPS from *S*. Enteritidis, even in the low dose mentioned above, which does not induce the symptoms of illness, was found to increase DA levels in the brain and the levels of some neuropeptides in lymph nodes (Figure 2). It also led to a reduction in the number of CD4 and CD8 T lymphocytes in the blood. It should be noted that, in animals, the terms subclinical and asymptomatic dose are synonymous (asymptomatic animals are subclinical), and both terms refer to a dose that does not cause any symptoms of illness [85]. Therefore, the subclinical dose of LPS from *S.* Enteritidis is the same as the asymptomatic dose of LPS from *S.* Enteritidis in animal models.

In light of studies on the role of the calcitonin gene-related peptide (CGRP) in protecting against *Salmonella* infection, as well as the changes in the neurochemical coding of selected neuropeptides in the wall of the porcine gallbladder and duodenum under the influence of an asymptomatic dose of *S.* Enteritidis LPS [74,75,83], the recent finding [86] that cGRP modulates microfold cells in mouse Peyer’s patches to protect *against S.* Typhimurium infection is very interesting. Furthermore, dorsal root ganglion (DRG) nociceptor neurons are able to directly sense and release CGRP for the maintenance of segmented filamentous bacteria colonization in the mouse ileum. Nociceptors can probably directly sense not only bacterial processes, such as the *S.* Typhimurium inflammation process, but have also been found to sense bacterial molecules, including LPSs [86]. However, the influence of LPSs derived from *S.* Enteritidis, *S.* Minnesota, and *S.* Typhimurium on the percentage of CGRP-positive neurons of the DRG from neuromers Th7 to L4 was not observed in the in vitro study. Nevertheless, the highest number of sensory neurons supplying the ileocecal valve (highly related to small intestinal bacterial overgrowth) showed immunoreactivity to CGRP [87]. This may be connected with neuroprotective and/or adaptive processes used to maintain homeostasis, but CGRP’s role in the intestines has not yet been fully elucidated. The differences in the levels of the neuropeptides between particular segments of the intestines suggest that the mechanisms of response to LPSs depend on particular segments of the intestines [76]. The mechanisms of the changes observed are not fully understood. These dysregulations may be associated with either the neurodegenerative or proinflammatory activity of LPS; however, taking into account the diverse (including neuroprotective) functions of particular neuropeptides, it is extremely difficult to explain the aforementioned effects.

Moreover, even seven days after the administration of asymptomatic LPS from *S.* Enteritidis, dysregulation of the key regulators of the hormonal axes, such as corticoliberin (corticotropin-releasing hormone, CRH), thyroliberin (thyrotropin-releasing hormone, TRH), and gonadoliberin (gonadotropin-releasing hormone, GnRH), and neuropeptides in the selected structures of the hypothalamus and the endocrine glands of the HPA (hypothalamus–pituitary–adrenal axis), HPT (hypothalamus–pituitary–thyroid axis), and HPO (hypothalamus–pituitary–ovary axis) axes are observed [78] (Figure 2). LPS from *S.* Enteritidis, which does not cause any symptoms of illness, can also induce changes in the levels of the neuropeptides in the spinal cord and DRG [79]. LPS from *S.* Enteritidis in a dose that does not produce noticeable symptoms also affects gene expression in the adrenal cortex and endometrium cells of domestic pigs. It has been confirmed that such an endotoxin in low concentrations (which does not cause disease symptoms) can induce changes in the transcriptome expression and modulate molecular mechanisms that condition the maintenance of homeostasis during a state resembling an asymptomatic carrier state. The RIG-I-like receptor signaling pathway may play a more important role than the TLR4 signaling pathway after administering an asymptomatic dose of *S.* Enteritidis LPS [81,82]. It should be pointed out that one report noted a subclinical (asymptomatic) dose of LPS obtained from *Escherichia coli* O55 in mice [88]. Lew et al. [88] demonstrated that, compared to a single administration of a subclinical dose of LPS from *Escherichia coli* O55, its subsequent administration caused a rise in mortality and cardiac fibrosis in mice. It should be emphasized that the authors mentioned above warn against using repeat doses of LPSs in humans due to a lack of information regarding the long-term consequences induced by this endotoxin.

In view of the above, the presence of LPS from *S.* Enteritidis in the body in an amount that does not induce any symptoms of illness may result in unknown long-term consequences associated with its action on the cells and biologically active substances in the nervous, immune, and endocrine systems [76,77,78,79].

## 6. Heterogeneity and Variability of Structures and the Diverse Activity of LPSs from Different Serotypes of *Salmonella* spp.

Despite many studies of the structural analysis of LPSs from various bacteria [89,90], knowledge is lacking on data on the comparison of clinical activity of LPSs from different strains isolated from symptomatic and asymptomatic hosts. The activity of lipopolysaccharides, often structurally diverse, has not been fully explained. A Gram-negative bacterial cell contains approximately 3.5 million LPS molecules on its surface [91], which, in order to achieve their biological effect, must be released from a bacterial cell. LPS can be released from the bacteria’s outer membrane and circulate as a free molecule. This phenomenon may occur during normal bacterial division or the abnormal growth of the outer membrane. Moreover, LPS is also released during the natural death of bacteria, but the release can also be induced by antibiotic treatment.

**Figure 3:**
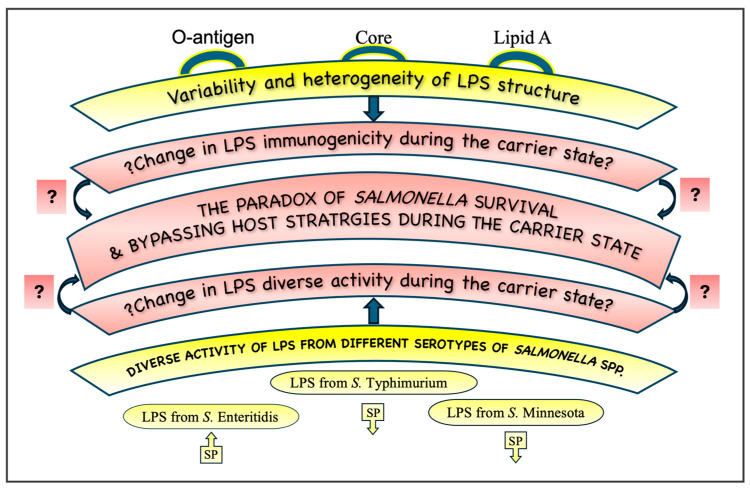
A possible explanation of the *Salmonella* paradox. The survival and bypassing of host defense strategies may be related to the change in LPS immunogenicity and activity during the carrier state. There are differences in LPS activity regarding the neuron neurochemical characterization within particular serotypes of *Salmonella* spp. (a low dose of LPS from *S.* Enteritidis induces an increase in substance P-positive neurons (SP), whereas LPSs from *S.* Minnesota and *S.* Typhimurium cause a decrease). The question mark indicates the need for the confirmation of the presented theory.

In terms of structure, an LPS molecule is characterized by the presence of the following three regions: the O-specific chain (O-antigen and O-specific polysaccharide), core oligosaccharide (constituting the central part of the LPS), and A lipid (the region anchoring the LPS in the outer membrane of the cellular wall). LPS detection through lipid A limits its ability to identify a bacterial species accurately because lipid A is highly conserved among species and serotypes. However, considerable structural differences in lipid A are the basis of altered host immune response. Lipid A can also change its structure in response to environmental factors. LPS might also be changed when a pathogen infects the host cell [92], and it seems very important to determine the molecular differences, i.e., in the profile and structure of LPS, between *Salmonella* strains isolated from carriers and patients in the active phase of the salmonellosis disease. Lipid A produced by *Salmonella* is highly immunogenic, while other bacteria, such as *Yersinia pestis*, produce an LPS of low immunogenicity in vivo [93]. Synthesizing an LPS of low immunogenicity can be a bacterial strategy to evade host immune response and increase intracellular survival. Hence, the paradox of *Salmonella* survival and the bypassing of host defense strategies may be related to the change in LPS immunogenicity during the carrier state (Figure 3). The O-antigen serves as a fingerprint to determine bacterial species and serotypes, and it is subject to change. Under certain conditions, smooth types of bacteria can mutate to rough strains (lacking O-antigen) to omit the energy-intensive synthesis of O-antigen, and their activity can then be changed. Strains that lack the O-antigen and the outer core are categorized as deep roughs. The O-antigen impairs *S.* Typhimurium LPS internalization in intestinal epithelial cells (not macrophages) and delays TLR4-mediated immune recognition [94]. It has been known for a long time that the O-antigen contributes to the evasion of host immune defenses, particularly the evasion of the complement cascade in *S.* Typhimurium [95]. *S.* Enteritidis requires an LPS with a long O-antigen to resist the complement system [96].

LPSs from different *Salmonella* serovars are differentially capable of activating TLR4. Differences in O-chain lengths can be important for bacterial adhesion, internalization, and virulence [97]. Structural and chain length differences in LPSs between the serotypes of *S.* Typhimurium are sufficient to drive different host immune responses by lipid regulation [98]. The few available study [99,100] results highlight the importance of serotype-specific effects in the LPS from *E. coli* in the inflammatory pathway. In an in vitro study, four LPS serotypes derived from *Escherichia coli* differed in their ability to trigger cytokine secretion by immune cells, especially at lower concentrations [100]. The differences in LPS activity regarding the neuron neurochemical characterization within particular serotypes of *Salmonella* spp. were observed in the in vitro studies. The low dose of LPSs derived from *S.* Enteritidis, *S*. Typhimurium, and *S*. Minnesota affected neuron phenotypes differently. The LPS from *S*. Enteritidis induces an increase in substance P-positive neurons, whereas LPSs both from *S*. Minnesota and *S*. Typhimurium cause a decrease (Figure 3). The *S*. Typhimurium LPS, unlike the *S*. Enteritidis LPS and *S*. Minnesota LPS, did not affect the immunoreactivity to galanin [87]. Taking the above into consideration, the activity of LPS may vary and may depend not only on the species [21,101,102] but also on the serotype of *Salmonella* spp. [87]. Pathogen heterogeneity is a key feature of pathogen populations that impacts host response [103].

An important element of the work on *Salmonella* and LPSs from *Salmonella* spp. is the analysis of their changeability. Future research strategies should take into account diverse LPS biological activity, which is dependent not only on the species but also on the serotypes of the bacteria from which they are derived. Bacteria are organisms changing their properties, and hence posing various threats, an example of which is the issue of antimicrobial resistance (AMR), with special consideration given to multiresistant bacterial strains. *Salmonella* spp. has a range of mechanisms to prevent the action of many antimicrobials used in clinical medicine. AMR is a very important issue related to the carrier state of *Salmonella* spp. Moreover, the relationship between certain virulence determinants, i.e., virulence genes and antibiotic resistance, occurs in *Salmonella* [7,104]. It seems that each serovar has acquired a unique set of genes that enable them to use distinct strategies to modulate the host immune response. Moreover, metagenomic analysis of *Salmonella* spp. genes allow for the determination of the expression of bacterial genes involved in LPS biosynthesis. Many genes are involved in LPS biosynthesis, and the relationship between changes in the biosynthesis gene expression and LPS concentration is not straightforward. For example, a rarely reported wzxE gene is involved in bacterial LPS biosynthesis by encoding a flippase that can flip the precursor of LPS across the membrane into the periplasm space. The wzxE from *Salmonella* can mediate the bacterial ability of adhesion and invasion in host cells, and can affect host immune responses by regulating O-antigen flipping [105].

The heterogeneity and variability of the LPS structures are connected with its detection, which makes it a challenging process. LPS detection, especially LPSs from pathogenic bacteria such as *Salmonella*, which can persist in an organism without causing symptoms of the disease, is a crucial but complicated task [106,107]. Assays for the detection of LPSs derived from given species and serotypes of bacteria are needed. It is also possible that low doses of LPS from *S.* Enteritidis, which do not cause the clinical symptoms of illness, may require detection and eradication, which may be of great importance, particularly regarding an asymptomatic carrier of *Salmonella* spp. The solution to problems of masked LPS and the phenomenon of low endotoxin recovery may be helpful in the discovery of new tests for bacterial serotypes of LPSs and improve the safety of human health [106,108].

## 7. Conclusions

Research involving the use of *Salmonella* and their endotoxins in the prevention and treatment of cancer and cancer metastasis is needed. Finding the balance between the therapeutic benefits and toxicity of *Salmonella* spp. and their LPSs can help develop anticancer therapy. Future studies on lipopolysaccharides from *Salmonella* spp. should consider their high variability and diversity. The activity of even low doses of LPS may vary and may depend on the species and serotype of the bacteria. Therefore, it is crucial to always report the origin of the lipopolysaccharide used in the research, including the serotype, in research publications. Moreover, the presence of LPS from *S*. Enteritidis in the body in an amount that does not induce any symptoms of illness may result in unknown long-term consequences associated with its action on the cells and biologically active substances of an organism. It is not ethical to conduct research involving the use of endotoxins on people if the long-term consequences of their use are not known. Therefore, research strategies and the pharmaceutical industry should consider the diverse biological activity of LPS, dependent on the sources of the bacteria and the long-term consequences of LPS used in various doses.

## References

[1] Maciel B.M., Rezende R.P., Sriranganathan N., Mares M. (2017). *Salmonella enterica*: Latency. Current Topics in Salmonella and Salmonellosis.

[2] Wang M., Qazi I.H., Wang L., Zhou G., Han H. (2020). *Salmonella* Virulence and Immune Escape. Microorganisms.

[3] Grote A., Piscon B., Manson A.L., Adani B., Cohen H., Livny J., Earl A.M., Gal-Mor O. (2024). Persistent *Salmonella* infections in humans are associated with mutations in the BarA/SirA regulatory pathway. Cell Host Microbe.

[4] Foster N., Tang Y., Berchieri A., Geng S., Jiao X., Barrow P. (2021). Revisiting Persistent *Salmonella* Infection and the Carrier State: What Do We Know?. Pathogens.

[5] Luk C.H., Valenzuela C., Gil M., Swistak L., Bomme P., Chang Y.-Y., Mallet A., Enninga J. (2021). *Salmonella* Enters a Dormant State within Human Epithelial Cells for Persistent Infection. PLoS Pathog..

[6] Rana S., Maurya S., Chadrasekhar H., Srikanth C.V. (2021). Molecular Determinants of Peaceful Coexistence versus Invasiveness of Non-Typhoidal *Salmonella*: Implications in Long-Term Side-Effects. Mol. Asp. Med..

[7] Sima C.M., Buzilă E.R., Trofin F., Păduraru D., Luncă C., Duhaniuc A., Dorneanu O.S., Nastase E.V. (2024). Emerging Strategies against Non-Typhoidal *Salmonella*: From Pathogenesis to Treatment. Curr. Issues Mol. Biol..

[8] Gasperini G., Massai L., De Simone D., Raso M.M., Palmieri E., Alfini R., Rossi O., Ravenscroft N., Kuttel M.M., Micoli F. (2024). O-Antigen Decorations in *Salmonella enterica* Play a Key Role in Eliciting Functional Immune Responses Against Heterologous Serovars in Animal Models. Front. Cell. Infect. Microbiol..

[9] Niehaus I. (2015). In Vivo Radiodetoxification of *Salmonella* Minnesota Lipopolysaccharides with Radio-Labeled Leucine Enkephalin Cures Sensory Polyneuropathy: A Case Report. Niger. Health J..

[10] Szponar B., Kraśnik L., Hryniewiecki T., Gamian A., Larsson L. (2003). Distribution of 3-Hydroxy Fatty Acids in Tissues after Intraperitoneal Injection of Endotoxin. Clin. Chem..

[11] Niehaus I., Lange J.H. (2003). Endotoxin: Is It an Environmental Factor in the Cause of Parkinson’s Disease?. Occup. Environ. Med..

[12] Yao Z., Mates J.M., Cheplowitz A.M., Hammer L.P., Maiseyeu A., Phillips G.S., Wewers M.D., Rajaram M.V., Robinson J.M., Anderson C.L. (2016). Blood-Borne Lipopolysaccharide Is Rapidly Eliminated by Liver Sinusoidal Endothelial Cells via High-Density Lipoprotein. J. Immunol..

[13] Ramachandran G. (2014). Gram-Positive and Gram-Negative Bacterial Toxins in Sepsis: A Brief Review. Virulence.

[14] Mohammadi B., Schedel I., Graf K., Teiwes A., Hecker H., Haameijer B., Scheinichen D., Piepenbrock S., Dengler R., Bufler J. (2008). Role of Endotoxin in the Pathogenesis of Critical Illness Polyneuropathy. J. Neurol..

[15] Deng I., Corrigan F., Zhai G., Zhou X.-F., Bobrovskaya L. (2020). Lipopolysaccharide Animal Models of Parkinson’s Disease: Recent Progress and Relevance to Clinical Disease. BBI Health.

[16] Hoban D.B., Connaughton E., Connaughton C., Hogan G., Thornton C., Mulcahy P., Moloney T.C., Dowd E. (2013). Further Characterisation of the LPS Model of Parkinson’s Disease: A Comparison of Intra-Nigral and Intra-Striatal Lipopolysaccharide Administration on Motor Function, Microgliosis and Nigrostriatal Neurodegeneration in the Rat. BBI Health.

[17] Huang B., Liu J., Ju C., Yang D., Chen G., Xu S., Zeng Y., Yan X., Wang W., Liu D. (2017). Licochalcone A Prevents the Loss of Dopaminergic Neurons by Inhibiting Microglial Activation in Lipopolysaccharide (LPS)-Induced Parkinson’s Disease Models. Int. J. Mol. Sci..

[18] Liu M., Bing G. (2011). Lipopolysaccharide Animal Models for Parkinson’s Disease. Park. Dis..

[19] Sharma N., Nehru B. (2015). Characterization of the Lipopolysaccharide Induced Model of Parkinson’s Disease: Role of Oxidative Stress and Neuroinflammation. Neurochem. Int..

[20] Perez-Pardo P., Dodiya H.B., Engen P.A., Forsyth C.B., Huschens A.M., Shaikh M., Voigt R.M., Naqib A., Green S.J., Kordower J.H. (2019). Role of TLR4 in the Gut-Brain Axis in Parkinson’s Disease: A Translational Study from Men to Mice. Gut.

[21] Anhê F.F., Barra N.G., Cavallari J.F., Henriksbo B.D., Schertzer J.D. (2021). Metabolic Endotoxemia Is Dictated by the Type of Lipopolysaccharide. Cell Rep..

[22] Manilla V., Di Tommaso N., Santopaolo F., Gasbarrini A., Ponziani F.R. (2023). Endotoxemia and Gastrointestinal Cancers: Insight into the Mechanisms Underlying a Dangerous Relationship. Microorganisms.

[23] Choi D.-Y., Liu M., Hunter R.L., Cass W.A., Pandya J.D., Sullivan P.G., Shin E.-J., Kim H.-C., Gash D.M., Bing G. (2009). Striatal Neuroinflammation Promotes Parkinsonism in Rats. PLoS ONE.

[24] Nguyen M.D., D’Aigle T., Gowing G., Julien J.P., Rivest S. (2004). Exacerbation of Motor Neuron Disease by Chronic Stimulation of Innate Immunity in a Mouse Model of Amyotrophic Lateral Sclerosis. J. Neurosci..

[25] Zhang X.Y., Cao J.B., Zhang L.M., Li Y.F., Mi W.D. (2015). Deferoxamine Attenuates Lipopolysaccharide-Induced Neuroinflammation and Memory Impairment in Mice. J. Neuroinflamm..

[26] Jain S., Dash P., Minz A.P., Satpathi S., Samal A.G., Behera P.K., Satpathi P.S., Senapati S. (2019). Lipopolysaccharide (LPS) enhances prostate cancer metastasis potentially through NF-κB activation and recurrent dexamethasone administration fails to suppress it in vivo. Prostate.

[27] Hawkesworth S., Moore S.E., Fulford A.J.C., Barclay G.R., Darboe A.A., Mark H., Nyan O.A., Prentice A.M. (2013). Evidence for Metabolic Endotoxemia in Obese and Diabetic Gambian Women. Nutr. Diabetes.

[28] Kallio K.A.E., Hätönen K.A., Lehto M., Salomaa V., Männistö S., Pussinen P.J. (2015). Endotoxemia, Nutrition, and Cardiometabolic Disorders. Acta Diabetol..

[29] Kang M., Edmundson P., Araujo-Perez F., McCoy A.N., Galanko J., Keku T.O. (2013). Association of Plasma Endotoxin, Inflammatory Cytokines and Risk of Colorectal Adenomas. BMC Cancer.

[30] Lee K.K., Yum K.S. (2012). Association of Endotoxins and Colon Polyp: A Case-Control Study. J. Korean Med. Sci..

[31] Pretorius E., Bester J., Kell D.B. (2016). A Bacterial Component to Alzheimer’s-Type Dementia Seen via a Systems Biology Approach That Links Iron Dysregulation and Inflammagen Shedding to Disease. JAD.

[32] Radilla-Vázquez R.B., Parra-Rojas I., Martínez-Hernández N.E., Márquez-Sandoval Y.F., Illades-Aguiar B., Castro-Alarcón N. (2016). Gut Microbiota and Metabolic Endotoxemia in Young Obese Mexican Subjects. Obes. Facts.

[33] Zhan X., Stamova B., Jin L.W., DeCarli C., Phinney B., Sharp F.R. (2016). Gram-Negative Bacterial Molecules Associate with Alzheimer Disease Pathology. Neurology.

[34] Zhao Y., Jaber V., Lukiw W.J. (2017). Secretory Products of the Human GI Tract Microbiome and Their Potential Impact on Alzheimer’s Disease (AD): Detection of Lipopolysaccharide (LPS) in AD Hippocampus. Front. Cell. Infect. Microbiol..

[35] Banks W.A., Gray A.M., Erickson M.A., Salameh T.S., Damodarasamy M., Sheibani N., Meabon J.S., Wing E.E., Morofuji Y., Cook D.G. (2015). Lipopolysaccharide-induced blood-brain barrier disruption: Roles of cyclooxygenase, oxidative stress, neuroinflammation, and elements of the neurovascular unit. J. Neuroinflamm..

[36] Banks W.A., Robinson S.M. (2010). Minimal Penetration of Lipopolysaccharide across the Murine Blood–Brain Barrier. BBI Health.

[37] Marques F., Sousa J.C., Coppola G., Falcao A.M., Rodrigues A.J., Geschwind D.H., Sousa N., Correia-Neves M., Palha J.A. (2009). Kinetic Profile of the Transcriptome Changes Induced in the Choroid Plexus by Peripheral Inflammation. J. Cereb. Blood Flow Metab..

[38] Marques F., Sousa J.C., Coppola G., Geschwind D.H., Sousa N., Palha J.A., Correia-Neves M. (2009). The Choroid Plexus Response to a Repeated Peripheral Inflammatory Stimulus. BMC Neurosci..

[39] Mughini-Gras L., Schaapveld M., Kramers J., Mooij S., Neefjes-Borst E.A., Pelt W.V., Neefjes J. (2018). Increased Colon Cancer Risk after Severe *Salmonella* Infection. PLoS ONE.

[40] Shanker E.B., Sun J. (2023). *Salmonella* Infection Acts as an Environmental Risk Factor for Human Colon Cancer. Cell Insight.

[41] Iyer P., Barreto S.G., Sahoo B., Chandrani P., Ramadwar M.R., Shrikhande S.V., Dutt A. (2016). Non-Typhoidal *Salmonella* DNA Traces in Gallbladder Cancer. Infect. Agents Cancer.

[42] Liu S., Li W., Chen J., Li M., Geng Y., Liu Y., Wu W. (2024). The Footprint of Gut Microbiota in Gallbladder Cancer: A Mechanistic Review. Front. Cell. Infect. Microbiol..

[43] Sarma J., Huda F., Naithani M., Kumar Singh S., Kumar N., Basu S., Rodrigo L. (2022). Role of Gut Microbiome and Enteric Bacteria in Gallbladder Cancer. Immunology of the GI Tract—Recent Advances.

[44] van Elsland D.M., Duijster J.W., Zhang J., Stévenin V., Zhang Y., Zha L., Xia Y., Franz E., Sun J., Mughini-Gras L. (2022). Repetitive Non-Typhoidal *Salmonella* Exposure Is an Environmental Risk Factor for Colon Cancer and Tumor Growth. Cell Rep. Med..

[45] Scanu T., Spaapen R.M., Bakker J.M., Pratap C.B., Wu L., Hofland I., Broeks A., Shukla V.K., Kumar M., Janssen H. (2015). *Salmonella* Manipulation of Host Signaling Pathways Provokes Cellular Transformation Associated with Gallbladder Carcinoma. Cell Host Microbe.

[46] Upadhayay A., Pal D., Kumar A. (2022). *Salmonella* Typhi Induced Oncogenesis in Gallbladder Cancer: Co-Relation and Progression. Adv. Cancer Biol. Metastasis.

[47] Nandi I., Aroeti B. (2023). Mitogen-Activated Protein Kinases (MAPKs) and Enteric Bacterial Pathogens: A Complex Interplay. Int. J. Mol. Sci..

[48] Pillay T.D., Hettiarachchi S.U., Gan J., Diaz-Del-Olmo I., Yu X.J., Muench J.H., Thurston T.L.M., Pearson J.S. (2023). Speaking the Host Language: How *Salmonella* Effector Proteins Manipulate the Host: This Article Is Part of the Bacterial Cell Envelopes Collection. Microbiology.

[49] Jiao Y., Zhang Y., Lin Z., Lu R., Xia Y., Meng C., Pan Z., Xu X., Jiao X., Sun J. (2020). *Salmonella* Enteritidis Effector AvrA Suppresses Autophagy by Reducing Beclin-1 Protein. Front. Immunol..

[50] Kushwaha M., Nukala V., Singh A.K., Makharia G.K., Mohan A., Kumar A., Dalal N. (2024). Emerging Implications of Bacterial Biofilm in Cancer Biology: Recent Updates and Major Perspectives. Gut Microbes Rep..

[51] Aljahdali N.H., Sanad Y.M., Han J., Foley S.L. (2020). Current Knowledge and Perspectives of Potential Impacts of *Salmonella enterica* on the Profile of the Gut Microbiota. BMC Microbiol..

[52] Yarahmadi A., Zare M., Aghayari M., Afkhami H., Jafari G.A. (2024). Therapeutic Bacteria and Viruses to Combat Cancer: Double-Edged Sword in Cancer Therapy: New Insights for Future. Cell Commun. Signal..

[53] Kocijancic D., Leschner S., Felgner S., Komoll R.-M., Frahm M., Pawar V., Weiss S. (2017). Therapeutic Benefit of *Salmonella* Attributed to LPS and TNF-α Is Exhaustible and Dictated by Tumor Susceptibility. Oncotarget.

[54] Chettab K., Fitzsimmons C., Novikov A., Denis M., Phelip C., Mathé D., Choffour P.A., Beaumel S., Fourmaux E., Norca P. (2023). A Systemically Administered Detoxified TLR4 Agonist Displays Potent Antitumor Activity and an Acceptable Tolerance Profile in Preclinical Models. Front. Immunol..

[55] Roe J.M., Seely K., Bussard C.J., Eischen Martin E., Mouw E.G., Bayles K.W., Hollingsworth M.A., Brooks A.E., Dailey K.M. (2023). Hacking the Immune Response to Solid Tumors: Harnessing the Anti-Cancer Capacities of Oncolytic Bacteria. Pharmaceutics.

[56] Yang Z., Zou L., Yue B., Hu M. (2023). *Salmonella typhimurium* May Support Cancer Treatment: A Review. ABBS.

[57] Zhao X., Xie N., Zhang H., Zhou W., Ding J. (2023). Bacterial Drug Delivery Systems for Cancer Therapy: “Why” and “How”. Pharmaceutics.

[58] Guo Y., Chen Y., Liu X., Min J.-J., Tan W., Zheng J.H. (2020). Targeted Cancer Immunotherapy with Genetically Engineered Oncolytic *Salmonella typhimurium*. Cancer Lett..

[59] Toso J.F., Gill V.J., Hwu P., Marincola F.M., Restifo N.P., Schwartzentruber D.J., Sherry R.M., Topalian S.L., Yang J.C., Stock F. (2002). Phase I Study of the Intravenous Administration of Attenuated *Salmonella typhimurium* to Patients with Metastaticmelanoma. J. Clin. Oncol..

[60] Uchugonova A., Zhao M., Zhang Y., Weinigel M., König K., Hoffman R.M. (2012). Cancer-Cell Killing By Engineered *Salmonella* Imaged By Multiphoton Tomography In Live Mice. Anticancer Res..

[61] Gniadek T.J., Augustin L., Schottel J., Leonard A., Saltzman D., Greeno E., Batist G. (2020). A Phase I, Dose Escalation, Single Dose Trial of Oral Attenuated *Salmonella typhimurium* Containing Human IL-2 in Patients with Metastatic Gastrointestinal Cancers. J. Immunother..

[62] Liang K., Zhang R., Luo H., Zhang J., Tian Z., Zhang X., Zhang Y., Ali M.K., Kong Q. (2021). Optimized Attenuated *Salmonella typhimurium* Suppressed Tumor Growth and Improved Survival in Mice. Front. Microbiol..

[63] Guan Y. (2021). ClyA Enhances LPS-Induced IL-1β Secretion in Human Macrophages through TLR4 and NLRP3 Signaling. J. Biol. Regul. Homeost. Agents.

[64] Tan W., Duong M.T.Q., Zuo C., Qin Y., Zhang Y., Guo Y., Hong Y., Zheng J.H., Min J.J. (2022). Targeting of Pancreatic Cancer Cells and Stromal Cells Using Engineered Oncolytic *Salmonella typhimurium*. Mol. Ther..

[65] Chen W., Zhu Y., Zhang Z., Sun X. (2022). Advances in *Salmonella typhimurium*-Based Drug Delivery System for Cancer Therapy. Adv. Drug Deliv. Rev..

[66] Liu X., Guo Y., Sun Y., Chen Y., Tan W., Min J.J., Zheng J.H. (2022). Comparison of Anticancer Activities and Biosafety Between *Salmonella enterica* Serovar Typhimurium ΔppGpp and VNP20009 in a Murine Cancer Model. Front. Microbiol..

[67] Barati M., Mirzavi F., Atabaki M., Bibak B., Mohammadi M., Jaafari M.R. (2022). A Review of PD-1/PD-L1 siRNA Delivery Systems in Immune T Cells and Cancer Cells. Int. Immunopharmacol..

[68] Chen P., Li Y., Wei P., Liang L., Li B., Cao Y., Han X., Wang Y., Duan X., Jia H. (2022). siRNA Targeting PD-L1 Delivered with Attenuated *Salmonella* Enhanced the Anti-Tumor Effect of Lenvatinib on Mice Bearing Hepatocellular carcinoma. Int. Immunopharmacol..

[69] Jia H., Wei P., Zhou S., Hu Y., Zhang C., Liang L., Li B., Gan Z., Xia Y., Jiang H. (2023). Attenuated *Salmonella* Carrying siRNA-PD-L1 and Radiation Combinatorial Therapy Induces Tumor Regression on HCC through T Cell-Mediated Immuno-Enhancement. Cell Death Discov..

[70] Marei H.E., Hasan A., Pozzoli G., Cenciarelli C. (2023). Cancer Immunotherapy with Immune Checkpoint Inhibitors (ICIs): Potential, Mechanisms of Resistance, and Strategies for Reinvigorating T Cell Responsiveness When Resistance is Acquired. Cancer Cell Int..

[71] Lasselin J., Schedlowski M., Karshikoff B., Engler H., Lekander M., Konsman J.P. (2020). Comparison of Bacterial Lipopolysaccharide-Induced Sickness Behavior in Rodents and Humans: Relevance for Symptoms of Anxiety and Depression. Neurosci. Biobehav. Rev..

[72] Kyvelidou C., Sotiriou D., Zerva I., Athanassakis I. (2018). Protection Against Lipopolysaccharide-Induced Immunosuppression by IgG and IgM. Shock.

[73] Engelhardt R., Mackensen A., Galanos C. (1991). Phase I trial of intravenously administered endotoxin (*Salmonella abortus equi*) in cancer patients. Cancer Res..

[74] Makowska K., Mikolajczyk A., Calka J., Gonkowski S. (2018). Neurochemical Characterization of Nerve Fibers in the Porcine Gallbladder Wall under Physiological Conditions and after the Administration of *Salmonella* Enteritidis Lipopolysaccharides (LPS). Toxicol. Res..

[75] Mikołajczyk A., Makowska K. (2017). Cocaine- and Amphetamine-Regulated Transcript (CART) Peptide in the Nerve Fibres of the Porcine Gallbladder Wall Under Physiological Conditions and after *Salmonella* Enteritidis Lipopolysaccharides Administration. Folia Morphol..

[76] Mikołajczyk A., Gonkowski S., Złotkowska D. (2017). Modulation of the Main Porcine Enteric Neuropeptides by a Single Low-Dose of Lipopolysaccharide (LPS) *Salmonella* Enteritidis. Gut Pathog..

[77] Mikołajczyk A., Złotkowska D. (2018). Neuroimmunological Implications of Subclinical Lipopolysaccharide from *Salmonella* Enteritidis. Int. J. Mol. Sci..

[78] Mikołajczyk A., Złotkowska D. (2019). Subclinical Lipopolysaccharide from *Salmonella* Enteritidis Induces Dysregulation of Bioactive Substances from Selected Brain Sections and Glands of Neuroendocrine Axes. Toxins.

[79] Mikołajczyk A., Złotkowska D. (2019). Subclinical Lipopolysaccharide from *Salmonella* Enteritidis Induces Neuropeptide Dysregulation in the Spinal Cord and the Dorsal Root Ganglia. BMC Neurosci..

[80] Otto F., Schmid P., Mackensen A., Wehr U., Seiz A., Braun M., Galanos C., Mertelsmann R., Engelhardt R. (1996). Phase II Trial of Intravenous Endotoxin in Patients with Colorectal and Non-Small Cell Lung Cancer. Eur. J. Cancer.

[81] Paukszto L., Mikolajczyk A., Jastrzebski J.P., Majewska M., Dobrzyn K., Kiezun M., Smolinska N., Kaminski T. (2020). Transcriptome, Spliceosome and Editome Expression Patterns of the Porcine Endometrium in Response to a Single Subclinical Dose of *Salmonella* Enteritidis Lipopolysaccharide. Int. J. Mol. Sci..

[82] Paukszto L., Mikolajczyk A., Szeszko K., Smolinska N., Jastrzebski J.P., Kaminski T. (2019). Transcription Analysis of the Response of the Porcine Adrenal Cortex to a Single Subclinical Dose of Lipopolysaccharide from *Salmonella* Enteritidis. Int. J. Biol. Macromol..

[83] Rytel L., Wojtkiewicz J., Snarska A., Mikołajczyk A. (2021). Changes in the Neurochemical Characterization of Enteric Neurons in the Porcine Duodenum After Administration of Low-Dose *Salmonella* Enteritidis Lipopolysaccharides. J. Mol. Neurosci..

[84] van Loon L.M., Stolk R.F., van der Hoeven J.G., Veltink P.H., Pickkers P., Lemson J., Kox M. (2020). Effect of Vasopressors on the Macro- and Microcirculation During Systemic Inflammation in Humans In Vivo. Shock.

[85] Rishniw M., White M.E., Mueller N. (2022). The Terms Asymptomatic and Subclinical Are the Same in the Veterinary Lexicon: A Critical Analysis. J. Am. Vet. Med. Assoc..

[86] Lai N.Y., Musser M.A., Pinho-Ribeiro F.A., Baral P., Jacobson A., Ma P., Potts D.E., Chen Z., Paik D., Soualhi S. (2020). Gut-Innervating Nociceptor Neurons Regulate Peyer’s Patch Microfold Cells and SFB Levels to Mediate *Salmonella* Host Defense. Cell.

[87] Mikołajczyk A., Kozłowska A., Gonkowski S. (2018). Distribution and Neurochemistry of the Porcine Ileocaecal Valve Projecting Sensory Neurons in the Dorsal Root Ganglia and the Influence of Lipopolysaccharide from Different Serotypes of *Salmonella* spp. on the Chemical Coding of DRG Neurons in the Cell Cultures. Int. J. Mol. Sci..

[88] Lew W.Y.W., Bayna E., Molle E.D., Dalton N.D., Lai N.C., Bhargava V., Mendiola V., Clopton P., Tang T. (2013). Recurrent Exposure to Subclinical Lipopolysaccharide Increases Mortality and Induces Cardiac Fibrosis in Mice. PLoS ONE.

[89] Fux A.C., Casonato Melo C., Michelini S., Swartzwelter B.J., Neusch A., Italiani P., Himly M. (2023). Heterogeneity of Lipopolysaccharide as Source of Variability in Bioassays and LPS-Binding Proteins as Remedy. Int. J. Mol. Sci..

[90] Maldonado R.F., Sá-Correia I., Valvano M.A. (2016). Lipopolysaccharide Modification in Gram-Negative Bacteria during Chronic Infection. FEMS Microbiol. Rev..

[91] Crinnion W.J., Pizzorno J.E. (2018). Clinical Environmental Medicine: Identification and Natural Treatment of Diseases Caused by Common Pollutants, Section II: The Toxicans.

[92] Stojkovic K., Szijártó V., Kaszowska M., Niedziela T., Hartl K., Nagy G., Lukasiewicz J. (2017). Identification of D-Galactan-III as Part of the Lipopolysaccharide of Klebsiella Pneumoniae Serotype O1. Front. Microbiol..

[93] Farhana A., Khan Y.S. (2024). Biochemistry, Lipopolysaccharide. StatPearls.

[94] Duerr C.U., Zenk S.F., Chassin C., Pott J., Gütle D., Hensel M., Hornef M.W. (2009). O-Antigen Delays Lipopolysaccharide Recognition and Impairs Antibacterial Host Defense in Murine Intestinal Epithelial Cells. PLoS Pathog..

[95] Murray G.L., Attridge S.R., Morona R. (2006). Altering the Length of the Lipopolysaccharide O Antigen Has an Impact on the Interaction of *Salmonella enterica* Serovar Typhimurium with Macrophages and Complement. J. Bacteriol..

[96] Krzyżewska-Dudek E., Dulipati V., Kapczyńska K., Noszka M., Chen C., Kotimaa J., Książczyk M., Dudek B., Bugla-Płoskońska G., Pawlik K. (2024). Lipopolysaccharide with Long O-Antigen Is Crucial for *Salmonella* Enteritidis to Evade Complement Activity and to Facilitate Bacterial Survival in Vivo in the *Galleria mellonella* Infection Model. Med. Microbiol. Immunol..

[97] Chessa D., Spiga L., De Riu N., Delaconi P., Mazzarello V., Ganau G., Rubino S. (2014). Lipopolysaccharides Belonging to Different *Salmonella* Serovars Are Differentially Capable of Activating Toll-Like Receptor 4. Infect. Immun..

[98] Cian M.B., Giordano N.P., Masilamani R., Minor K.E., Dalebroux Z.D. (2019). *Salmonella enterica* Serovar Typhimurium Uses PbgA/YejM to Regulate Lipopolysaccharide Assembly During Bacteremia. Infect. Immun..

[99] Migale R., Herbert B.R., Lee Y.S., Sykes L., Waddington S.N., Peebles D., Hagberg H., Johnson M.R., Bennett P.R., MacIntyre D.A. (2015). Specific Lipopolysaccharide Serotypes Induce Differential Maternal and Neonatal Inflammatory Responses in a Murine Model of Preterm Labor. AJP.

[100] Vermeire B., Walsh M., Cox E., Devriendt B. (2024). The Lipopolysaccharide Structure Affects the Detoxifying Ability of Intestinal Alkaline Phosphatases. BMC Vet. Res..

[101] Pieterse E., Rother N., Yanginlar C., Hilbrands L.B., Van Der Vlag J. (2016). Neutrophils Discriminate between Lipopolysaccharides of Different Bacterial Sources and Selectively Release Neutrophil Extracellular Traps. Front. Immunol..

[102] Pulendran B., Kumar P., Cutler C.W., Mohamadzadeh M., Van Dyke T., Banchereau J. (2001). Lipopolysaccharides from Distinct Pathogens Induce Different Classes of Immune Responses In Vivo. J. Immun..

[103] Avraham R., Haseley N., Brown D., Penaranda C., Jijon H.B., Trombetta J.J., Satija R., Shalek A.K., Xavier R.J., Regev A. (2015). Pathogen Cell-to-Cell Variability Drives Heterogeneity in Host Immune Responses. Cell.

[104] Higgins D., Mukherjee N., Pal C., Sulaiman I.M., Jiang Y., Hanna S., Dunn J.R., Karmaus W., Banerjee P. (2020). Association of Virulence and Antibiotic Resistance in *Salmonella*—Statistical and Computational Insights into a Selected Set of Clinical Isolates. Microorganisms.

[105] Wang K.C., Huang C.H., Chang P.R., Huang M.T., Fang S.B. (2020). Role of wzxE in *Salmonella typhimurium* Lipopolysaccharide Biosynthesis and Interleukin-8 Secretion Regulation in Human Intestinal Epithelial Cells. Microbiol. Res..

[106] Dardelle F., Phelip C., Darabi M., Kondakova T., Warnet X., Combret E., Juranville E., Novikov A., Kerzerho J., Caroff M. (2024). Diversity, Complexity, and Specificity of Bacterial Lipopolysaccharide (LPS) Structures Impacting Their Detection and Quantification. Int. J. Mol. Sci..

[107] Virzì G.M., Mattiotti M., De Cal M., Ronco C., Zanella M., De Rosa S. (2022). Endotoxin in Sepsis: Methods for LPS Detection and the Use of Omics Techniques. Diagnostics.

[108] Gorman A., Golovanov A.P. (2022). Lipopolysaccharide Structure and the Phenomenon of Low Endotoxin Recovery. Eur. J. Pharm. Biopharm..

